# Neoadjuvant chemotherapy with dose dense MVAC is associated with improved survival after radical cystectomy compared to other cytotoxic regimens: A tertiary center experience

**DOI:** 10.1371/journal.pone.0259526

**Published:** 2021-11-03

**Authors:** Artur Lemiński, Krystian Kaczmarek, Tomasz Byrski, Marcin Słojewski

**Affiliations:** 1 Department of Urology and Urological Oncology, Pomeranian Medical University, Szczecin, Poland; 2 Department of Oncology and Chemotherapy, Pomeranian Medical University, Szczecin, Poland; Kalinga Institute of Medical Sciences, INDIA

## Abstract

**Introduction:**

Neoadjuvant chemotherapy has become standard of care for cisplatin-eligible patients with muscle-invasive bladder cancer qualified to radical cystectomy, providing a modest increase in 5-year overall survival rate. Several regimens are being employed for neoadjuvant treatment, largely because of their efficacy in metastatic setting. There is however a scarcity of evidence on the optimal cytotoxic regimen for neoadjuvant chemotherapy.

**Objectives:**

We evaluated the efficacy of different protocols of neoadjuvant chemotherapy amongst patients who underwent radical cystectomy at our institution.

**Methods:**

This is a single-center, retrospective, observational study including a cohort of 220 patients who underwent radical cystectomy between 2014 and 2020. The neoadjuvant chemotherapy cohort included 79 patients and was compared to the cohort of historical controls including 141 patients operated prior to routine administration of neoadjuvant chemotherapy and those who opted for upfront surgery.

**Results:**

Administration of neoadjuvant chemotherapy decreased the risk of overall and cancer-specific mortality HR = 0.625 (95% CI 0.414–0.944), p = 0.025 and HR = 0.579 (95% CI 0.348–0.964), p = 0.036. Rates of downstaging, complete responses, lymph node metastasis, extravesical extension and positive surgical margins significantly favored neoadjuvant chemotherapy. Out of cytotoxic regimens, dose-dense MVAC and gemcitabine-cisplatin were similarly efficacious providing 46.9% and 50% of downstaging to <ypT2N0 respectively, including 30.6% and 25% of complete remissions. However, only dose-dense MVAC was associated with reduction of all-cause and cancer specific mortality risk HR = 0.385 (95% CI 0.214–0.691) p = 0.001 and HR = 0.336 (95% CI 0.160–0.703), p = 0.004 respectively.

**Conclusions:**

Our study implies that neoadjuvant chemotherapy with subsequent radical cystectomy provides significant improvement over upfront surgery in locoregional control and long-term prognosis of muscle-invasive bladder cancer. The urologic community should strive to maximize utilization of neoadjuvant chemotherapy, yet further research, including randomized control trials, is needed to validate superiority of dose-dense MVAC as the preferred regimen for cisplatin-eligible patients.

## Introduction

Muscle-invasive bladder cancer (MIBC) is an aggressive malignancy requiring prompt diagnostic evaluation and a multidisciplinary approach to treatment. Neoadjuvant chemotherapy (NAC) and subsequent radical cystectomy (RC) with pelvic lymph node dissection have well established oncological outcomes and remain the mainstay of care for patients, who are fit for cisplatin and surgery. According to available data, administration of cisplatin-based NAC provides an 8% increase in 5 year overall survival (OS) after RC, however the optimal regimen of NAC has not been established [1, 2]. Traditionally, two cytotoxic regimens: methotrexate, vinblastine, doxorubicin, cisplatin (MVAC) and gemcitabine-cisplatin (GC) have been commonly employed for NAC, largely as a consequence of their proven efficacy in the metastatic setting. In the neoadjuvant scenario both regimens were shown to perform similarly in terms of complete pathologic responses, however the GC regimen has been associated with inferior OS after cystectomy [3]. More recently, a dose dense variant of MVAC (ddMVAC) regimen has been adopted in the neoadjuvant setting, based on its conveniently short treatment duration, higher efficacy and more acceptable toxicity profile [4, 5].

We sought to evaluate the efficacy of different NAC regimens within the cohort of patients with MIBC, who underwent RC at our institution.

## Material and methods

This single-center non-randomized clinical follow-up study was exempt from further review by the Institutional Review Board (Bioethical Committee) of the Pomeranian Medical University, Szczecin, Poland (protocol number KB-0012/95/08/2021/Z) and was conducted with respect to regulations set forth by the Declaration of Helsinki. Patients involved provided written informed consent for scientific use of anonymized treatment data collected at the time of their hospital stay. We included 264 consecutive patients with pathologically confirmed MIBC who underwent RC between 2014 and 2020. Clinical, pathological and survival data of all cases have been prospectively collected in a dedicated database. The NAC is being consistently offered to patients with MIBC qualified to RC at our institution since 2017, and the uptake of NAC has been progressively increasing in following years. We identified 88 patients who received NAC before commencing to RC. The control group of 176 patients included those who underwent RC prior to 2017, when NAC was not routinely administered, and those operated afterwards, who opted for primary RC. We excluded patients with metastatic disease who underwent palliative cystectomy, partial bladder resections, patients with previous pelvic irradiation, with non-urothelial tumors and those with incomplete data (9 from the NAC group, 35 from controls). We evaluated the efficacy of NAC by comparing pathologic stage distribution after RC amongst patients receiving different chemotherapy regimens against chemo-naïve controls. We analyzed downstaging to node-negative, non-muscle invasive disease (<ypT2N0), which included pT0, pTa, pT1 and CIS, along with complete remission rates (ypT0N0—CR) for each regimen. Furthermore, we performed analysis of overall and cancer specific survival (CSS) in both study groups. Data were checked for internal consistency. Descriptive statistics including mean ± standard deviation (SD) and median (interquartile range—IQR) were provided for normally distributed and skewed data, respectively. Proportion was used for categorical variables. Univariable and multivariable logistic regression was performed to assess predicting factors for downstaging and complete remission. The survival probabilities over time were demonstrated with Kaplan–Meier survival estimates and univariate Cox models. Survival curves of different groups were compared using the log-rank test. Multivariable Cox proportional hazards models were applied to examine the impact of prognostic factors on OS and CSS. These included age at the RC, gender, severity of comorbidities reflected by American Society of Anesthesiologists (ASA) score, clinical stage, and chemotherapy regimen. Additionally, multivariable competing risk regression model proposed by Fine and Gray was implemented to model time from surgery to cancer specific death, considering deaths from other causes as competing events [6]. To control bias resulting from the retrospective nature of study and non-random assignment to treatment arms, propensity score matching was performed. Propensity scores were used to evaluate adjusted odds ratios (OR) of downstaging to < ypT2N0 and pT0N0 along with hazard ratios (HR) of survival for NAC regimens against non-NAC controls. Backward stepwise elimination was used to find reduced models that best explain the data. Assessing the proportional hazards assumption of the final multivariable models was carried out using scaled Schoenfeld residuals with time, to test for independence between residuals and time. The results of Cox proportional hazard models and competing risk regression model are presented as HR along with their 95% confidence intervals. All statistical tests were two-sided, and p-values < 0.05 were considered statistically significant. Tests were performed with Statistica software, version 13.5 (StatSoft, Inc., Tulsa, OK, USA) and R (version 3.5.1) and RStudio (version 1.4.1717) with R packages *survival*, *cmprsk* [7].

## Results

A cohort of 220 patients was included in final analysis, 79 patients constituted the NAC group, 141 were included as controls. Median follow-up durations were 19.3 months (IQR 9.1–54.17) and 24.7 months (IQR 12.6–39.7) respectively. There were no significant differences regarding age, gender, estimated glomerular filtration rate at baseline and distribution of ASA scores. The NAC exposure between 2014 and 2016 was limited to 11.4% of patients and increased to an average of 55.7% between 2017 and 2020; p < 0.001. Within the NAC cohort 49 (62%) patients received ddMVAC regimen, 24 (30.4%)–gemcitabine-cisplatin and 6 (7.6%)–gemcitabine-carboplatin. Compared to controls, NAC group had favorable clinical and pathological stage distribution, with higher proportion of pT0N0 (29.1 vs 9.9%) and lower incidence of extravesical disease (34.2 vs 56.7%), p<0.001. Furthermore, patients who received NAC had markedly lower incidence of positive surgical margins (PSM—2.53 vs 19.86%), p<0.001 and fewer had lymph node metastasis (17.7 vs 37.6%), p<0.001. The characteristics of study population are summarized in Table 1.

**Table 1:** Baseline patients’ characteristics and distribution of outcomes.

	neoadjuvant chemotherapy			distribution of outcomes	
Variable	No NAC	NAC	*P* value	cancer deaths	non-cancer deaths
Totals, No.	141	79		80	38
Age, Mean (SD), Years	66.75 (8.29)	66.75 (6.73)	0.996	67.83 (8.14)	68.29 (8.18)
Sex, No. (%)			0.832		
Female	31 (21.99)	16 (20.25)		14 (17.50)	11 (28.95)
Male	110 (78.01)	63 (79.75)		66 (82.50)	27 (71.05)
ASA score, No. (%)			0.383		
1	10 (7.09)	4 (5.06)		6 (7.50)	1 (2.63)
2	105 (74.47)	56 (70.89)		57 (71.25)	25 (65.79)
3	26 (18.44)	18 (22.78)		16 (20.00)	12 (31.58)
4	0 (0.00)	1 (1.27)		1 (1.25)	0 (0.00)
Preoperative eGFR, No. (%)			0.941		
≥90	37 (26.24)	16 (20.25)		17 (21.25)	4 (10.53)
60–89	53 (37.59)	38 (48.10)		34 (42.50)	14 (36.84)
30–59	40 (28.37)	22 (27.85)		23 (28.75)	15 (39.47)
≤29	11 (7.80)	3 (3.80)		6 (7.50)	5 (13.16)
Clinical T stage, No. (%)			0.018		
cT2	73 (51.77)	54 (68.35)		38 (47.50)	15 (39.47)
cT3	51 (36.17)	23 (29.11)		33 (41.25)	17 (44.74)
cT4	17 (12.06)	2 (2.53)		9 (11.25)	6 (15.79)
Pathological T stage, No. (%)			0.001		
pT0	14 (9.93)	23 (29.11)		4 (5.00)	6 (15.79)
pTis	1 (0.71)	1 (1.27)		0 (0.00)	1 (2.63)
pTa	2 (1.42)	2 (2.53)		0 (0.00)	2 (5.26)
pT1	20 (14.18)	11 (13.92)		6 (7.50)	5 (13.16)
pT2	24 (17.02)	15 (18.99)		7 (8.75)	5 (13.16)
pT3	43 (30.50)	16 (20.25)		35 (43.75)	11 (28.95)
pT4	37 (26.24)	11 (13.92)		28 (35.00)	8 (21.05)
Pathological N stage, No. (%)			0.015		
pN0	88 (62.41)	65 (82.28)		38 (47.50)	23 (60.53)
pN+	53 (37.59)	14 (17.72)		42 (52.50)	15 (39.47)
Surgical margin, No. (%)			0.033		
Negative	113 (80.14)	77 (97.47)		64 (80.00)	29 (76.32)
Positive	28 (19.86)	2 (2.53)		16 (20.00)	9 (23.68)
Chemotherapy regimen, No. (%)					
None	141 (100.00)	NA		60 (75.00)	28 (73.68)
ddMVAC	NA	49 (62.03)		8 (10.00)	5 (13.16)
Gemcitabine-cisplatin	NA	24 (30.38)		9 (11.25)	4 (10.53)
Gemcitabine-carboplatin	NA	6 (7.59)		3 (3.75)	1 (2.63)
Year of treatment, No. (%)					
2014–2016	89 (63.12)	10 (12.66)		47 (58.75)	17 (44.74)
2017–2020	52 (36.88)	69 (87.34)		33 (41.25)	21 (55.26)
Follow-up, Months			0.579		
Median (IQR)	18.33 (7.80–51.70)	19.33 (9.67–33.10)		13.25 (8.05–21.82)	6.58 (0.77–13.97)

**Fig 1:**
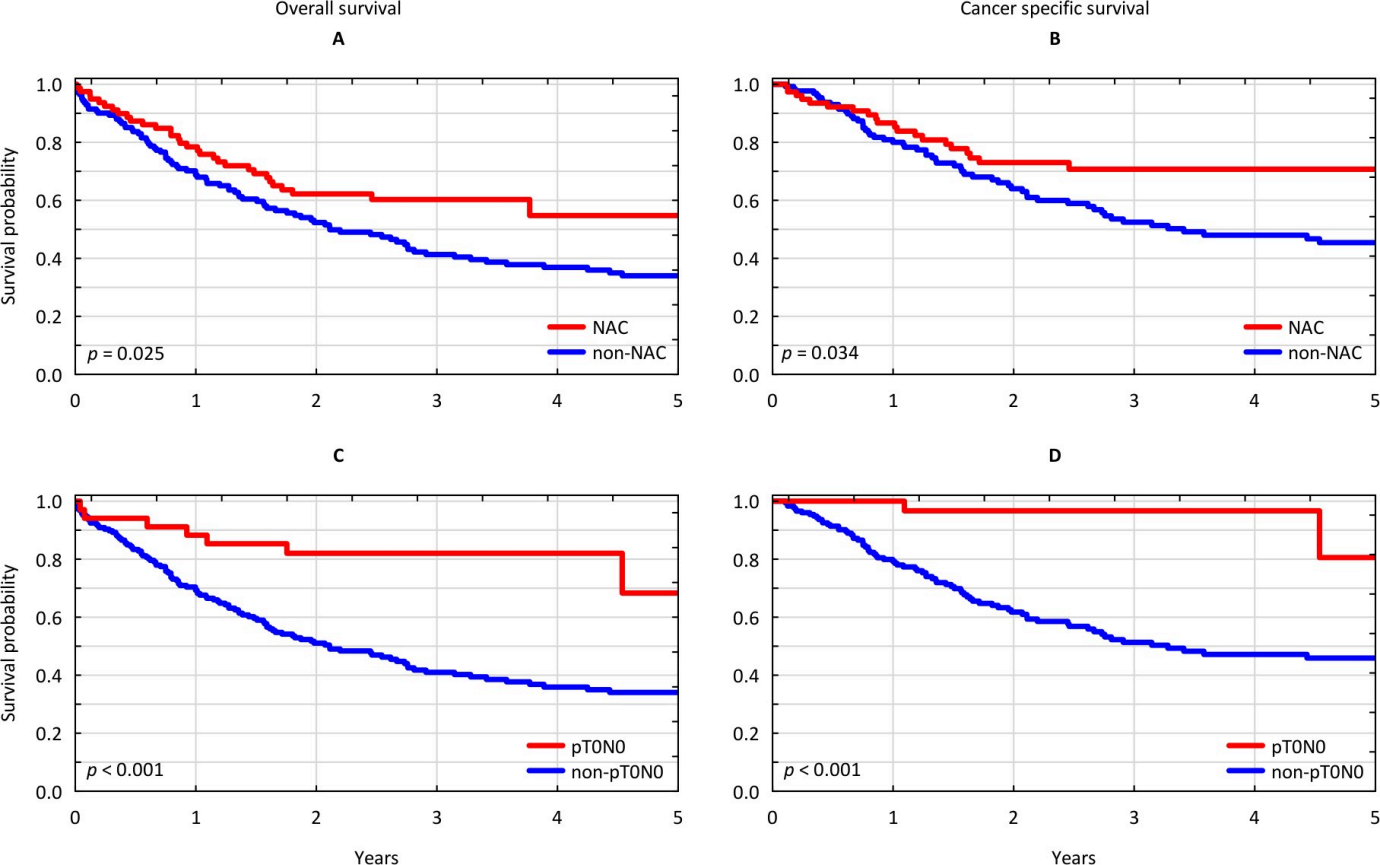
Survival analysis. (A) Kaplan-Meier curves for overall survival stratified by neoadjuvant chemotherapy. (B) Kaplan-Meier curves for cancer specific survival stratified by neoadjuvant chemotherapy. (C) Kaplan-Meier curves for overall survival for patients with complete response (pT0N0) vs those without complete responses (non-pT0N0). (D) Kaplan-Meier curves for cancer specific survival for patients with complete response (pT0N0) vs those without complete responses (non-pT0N0). NAC: neoadjuvant chemotherapy.

Above all, NAC provided significant reduction in risk of all-cause and cancer-specific mortality HR = 0.625 (95% confidence interval [CI] 0.414–0.944), p = 0.025 and HR = 0.579 (95% CI 0.348–0.964), p = 0.036, respectively. Consequently, patients from the NAC group benefited in terms of survival with 3-year OS and CSS of 60.27% (95% CI 49.05%-71.48%) and 70.72% (95% CI 59.67%-81.78%) respectively, compared to 41.34% (95% CI 32.79%-49.89%) and 52.47% (95% CI 42.94%-62.00%) with upfront surgery, p = 0.025 and 0.034 respectively (Fig 1A and 1B).

A subset of patients who achieved complete response with NAC (pT0N0) experienced superior survival outcome after RC with 3-year OS and CSS of 82.01% (95% CI 68.95%-95.08%) and 96.67% (95% CI 90.24%-100.00%) respectively, compared to 41.02% (95% CI 33.43%-48.61%) and 51.31% (95% CI 42.9%-59.71%) in the remaining population p<0.001 (Fig 1C and 1D). Among the cytotoxic regimens used the ddMVAC and gemcitabine-cisplatin were similarly effective in terms of downstaging rates. We observed disease downstaging to < ypT2N0 in 23 (46.95%) of patients treated with ddMVAC OR = 2.784 (95% CI 1.409–5.500); p = 0.003, including CR in 15 (30.61%) of patients, OR = 4.344 (95% CI 1.888–9.996); p = 0.001. The gemcitabine-cisplatin provided disease downstaging to < ypT2N0 in 12 (50%) of cases OR = 3.147 (95% CI 1.294–7.651), p = 0.011, including CR in 6 (25%) of patients, OR = 3.282 (95% CI 1.108–9.722), p = 0.032 (S1 and S2 Tables). Nonetheless, only patients who were received ddMVAC experienced significant reduction in risk of all-cause and cancer-specific mortality, HR = 0.385 (95% CI 0.214–0.691), p = 0.001 and 0.336 (95% CI 0.160–0.703), p = 0.004 respectively (S3 and S4 Tables). We validated these findings using competing risk regression model, in which only ddMVAC regimen consistently showed improvement over upfront surgery HR = 0.366 (0.176–0.759) P = 0.007 (Table 2 and S5 Table).

**Table 2:** Univariable, multivariable and Propensity-Weighted Regression analysis of factors predicting downstaging (<pT2N0), complete (pT0N0) pathological response and survival outcomes.

Chemotherapy regimen	No. (%)	Univariable	*P* Value	Multivariable^a^	*P* Value	Propensity score^b^	*P* Value
**Downstaged**		OR (95% CI)		OR (95% CI)		OR (95% CI)	
None	34 (24.11)	1 [Reference]		1 [Reference]		1 [Reference]	
ddMVAC	23 (46.94)	2.784 (1.409–5.500)	0.003	2.875 (1.441–5.737)	0.003	2.241 (1.010–4.976)	0.047
Gemcitabine-cisplatin	12 (50.00)	3.147 (1.294–7.651)	0.011	3.419 (1.379–8.476)	0.008	2.465 (0.889–6.838)	0.083
Gemcitabine-carboplatin	1 (16.67)	0.629 (0.071–5.576)	0.677	0.556 (0.062–4.964)	0.600	0.419 (0.040–4.381)	0.468
All regimens	36 (45.57)	2.635 (1.464–4.740)	0.001	2.478 (1.308–4.694)	0.005	1.963 (1.001–3.852)	0.049
**Complete response**		OR (95% CI)		OR (95% CI)		OR (95% CI)	
None	13 (9.22)	1 [Reference]		1 [Reference]		1 [Reference]	
ddMVAC	15 (30.61)	4.344 (1.888–9.996)	0.001	4.344 (1.888–9.996)	0.001	4.538 (1.694–12.158)	0.003
Gemcitabine-cisplatin	6 (25.00)	3.282 (1.108–9.722)	0.032	3.282 (1.108–9.722)	0.032	3.429 (1.026–11.458)	0.045
Gemcitabine-carboplatin	0 (0.00)	0.000 (0.000–0.000)	0.998	0.000 (0.000–0.000)	0.998	0.000 (0.000–0.000)	0.998
All regimens	21 (26.58)	3.565 (1.670–7.608)	0.001	3.433 (1.18–7.761)	0.003	3.724 (1.480–9.370)	0.005
**All-cause Cox PH model**		HR (95% CI)		HR (95% CI)		HR (95% CI)	
None	NA	1 [Reference]		1 [Reference]		1 [Reference]	
ddMVAC	NA	0.385 (0.214–0.691)	0.001	0.404 (0.218–0.749)	0.004	0.452 (0.242–0.842)	0.012
Gemcitabine-cisplatin	NA	1.062 (0.603–1.870)	0.834	1.060 (0.600–1.873)	0.841	1.080 (0.590–1.974)	0.804
Gemcitabine-carboplatin	NA	1.471 (0.539–4.015)	0.452	1.375 (0.488–3.872)	0.547	1.461 (0.498–4.285)	0.489
All regimens	NA	0.625 (0.414–0.944)	0.025	0.663 (0.433–1.016)	0.059	0.694 (0.437–1.103)	0.112
**Cause-specific Cox PH model**		HR (95% CI)		HR (95% CI)		HR (95% CI)	
None	NA	1 [Reference]		1 [Reference]		1 [Reference]	
ddMVAC	NA	0.336 (0.160–0.703)	0.004	0.325 (0.147–0.722)	0.006	0.297 (0.129–0.681)	0.004
Gemcitabine-cisplatin	NA	1.010 (0.500–2.038)	0.978	1.009 (0.497–2.046)	0.981	0.883 (0.422–1.849)	0.741
Gemcitabine-carboplatin	NA	1.661 (0.520–5.309)	0.392	1.694 (0.508–5.654)	0.391	1.360 (0.382–4.837)	0.635
All regimens	NA	0.579 (0.348–0.964)	0.036	0.593 (0.350–1.004)	0.052	0.536 (0.306–0.940)	0.030
**Competing risk regression model**		HR (95% CI)		HR (95% CI)		HR (95% CI)	
None	NA	1 [Reference]		1 [Reference]		1 [Reference]	
ddMVAC	NA	0.366 (0.176–0.759)	0.007	0.321 (0.145–0.710)	0.005	0.287 (0.126–0.653)	0.003
Gemcitabine-cisplatin	NA	1.004 (0.479–2.105)	0.990	1.016 (0.481-.2.150)	0.970	0.876 (0.405–1.894)	0.740
Gemcitabine-carboplatin	NA	1.929 (0.504–7.380)	0.340	1.968 (0.527–7.360)	0.310	1.704 (0.464–6.253)	0.420
All regimens	NA	0.618 (0.371–1.030)	0.065	0.630 (0.363–1.090)	0.100	0.523 (0.297–0.919)	0.024

CI: confidence interval; ddMVAC: dose-dense methotrexate, vinblastine, doxorubicin and cisplatin; HR: hazard ratio; NA: not applicable; OR: odds ratio; PH: proportional hazard.

^a^ Backward stepwise regression.

^b^ Weighted for age, gender, severity of comorbidities reflected by American Society of Anesthesiologists (ASA) score, estimated glomerular filtration rate (eGFR) and clinical T stage histology.

We found no significant reduction in risk of all-cause or cancer-specific death with gemcitabine-cisplatin regimen HR = 1.062 (95% CI 0.603–1.870); p = 0.834 and 1.010 (95% CI 0.500–2.038) respectively. Six patients received the gemcitabine-carboplatin regimen with unsatisfactory results. There were no complete responses, and only one patient experienced downstaging. Expectedly, no influence on risk of death was observed.

**Fig 2:**
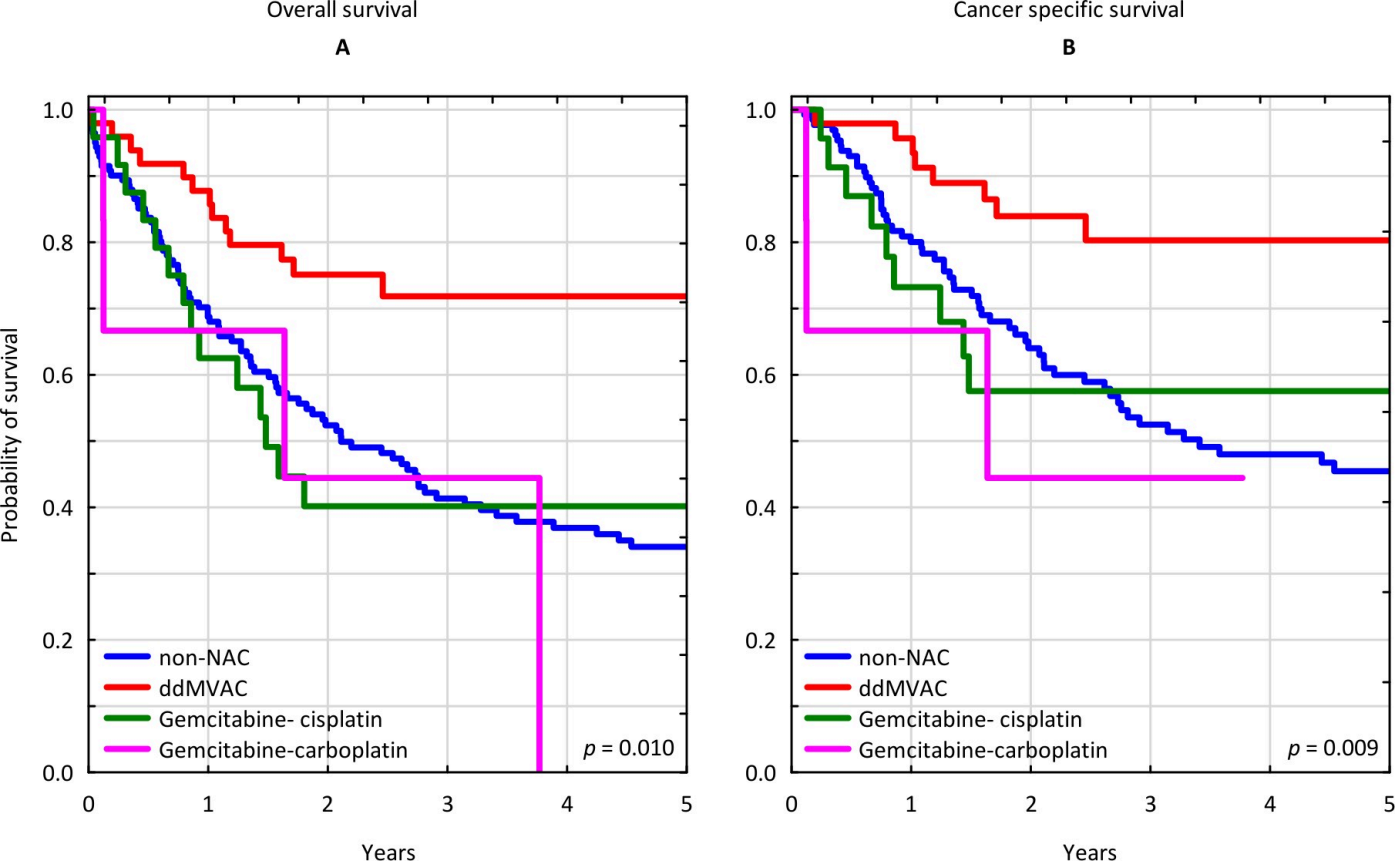
Survival analysis stratified by neoadjuvant chemotherapy regimen. (A) Kaplan-Meier curves for overall survival stratified by neoadjuvant chemotherapy regimen. (B) Kaplan-Meier curves for cancer specific survival stratified by neoadjuvant chemotherapy regimen. ddMVAC: dose-dense methotrexate, vinblastine, doxorubicin and cisplatin; NAC: neoadjuvant chemotherapy.

Patients who received ddMVAC regimen had a 3-year OS of 71.46% (95% CI 58.06–84.85%), which was significantly higher than in those, who received gemcitabine-cisplatin, and in patients treated with upfront RC: 40.18% (95% CI 20.15–60.21), p = 0.005 and 41.34% (95% CI 32.79–49.89%), p = 0.01 respectively (Fig 2A).

Furthermore, the ddMVAC protocol provided a favorable 3-year CSS of 80.07% (95% CI 67.38–100.00%), surpassing the gemcitabine-cisplatin protocol with 3-yr CSS of 57.54% (95% CI 36.18–78.89%) and upfront RC with 3-yr CSS of 52.47% (95% CI 42.94–62.00%), p = 0.009; (Fig 2B).

## Discussion

Neoadjuvant chemotherapy remains the standard of care for cisplatin-eligible patients with MIBC qualified to RC. It provides local downstaging of tumor in responders, facilitating radical resection of the bladder. Our study corroborated favorable pathologic stage migration within the NAC cohort, with downstaging to less than ypT2N0 in nearly half of patients. Moreover, 30.6% and 25% of patients achieved complete remissions with two most common chemotherapy regimens: ddMVAC and gemcitabine-cisplatin respectively with no significant differences between the two. These data are consistent with several reports on NAC published to date [1, 3, 4, 8–10]. One of the few studies challenging the downstaging rate of NAC by Weight et al. was conducted in a small patient cohort, utilized non-standard chemotherapy in 17% of patients, and analysis of response was hindered by a median RC delay exceeding 200 days [11].

In our contemporary series, downstaging of bladder cancer resulting from NAC caused nearly eight-fold decrease in PSM rate after RC, undoubtedly improving local control of disease. Similar data on decreasing incidence of PSM with increasing uptake of NAC were reported by Almassi et al. from Memorial Sloan Kettering Cancer Center, who found a similar PSM rate of 2.5% in their recent cystectomy patients, out of whom 57% received NAC [12].

There is now a general agreement on positive influence of NAC on local disease control, however debate is ongoing on magnitude of survival benefit achievable with combination therapy. Some recently published series failed to demonstrate a positive influence of NAC on the long term prognosis after RC, thereby fueling debate on existing treatment paradigm [9, 13, 14]. Our current analysis revealed a 42 per cent reduction in risk of cancer-specific death and 18.25% increase in 3-year CSS in patients who received NAC and underwent RC, compared to controls who underwent an upfront RC and adjuvant chemotherapy in case of lymph-node metastasis. The latter approach had been employed in our department for several decades and failed to improve long-term prognosis, producing marginal stage migration over the years [15, 16]. The distinctive feature of our series is inclusion of consecutive patients treated over the period of transition from the upfront cystectomy approach (2014–2016) to the multidisciplinary NAC-based approach (2017–2020), hence the clinical characteristics of both study arms are well matched. This, together with close follow-up of our cohort, made a head-to-head comparison of both treatment strategies feasible. Additionally, to the best of our knowledge, presented study is among the few in the literature to provide cancer-specific survival outcomes of different NAC protocols.

Findings from our series strongly endorse the idea of NAC and indicate its more profound influence on survival than reported in available literature. Data from recent systematic review and meta-analysis by Yin et al. show a 5-year OS benefit of NAC in range of 8%, while the study from Russel et. al. revealed a 10% improvement in 5-year OS in series of 944 RC patients from Swedish nationwide database [3, 17]. There are some plausible explanations of the aforementioned discrepancy. The majority of our NAC group received a ddMVAC regimen, an optimized derivative of the classical MVAC, featuring double dose-intensity of cisplatin and doxorubicin, along with two-thirds reduction of methotrexate and vinblastine. Hematological toxicity is managed with administration of pegfilgrastim in each cycle [18]. In our experience, this regimen proved well suited for NAC allowing delivery of increased dosage of cisplatin and doxorubicin over a shortened two weeks-per-cycle schedule, minimizing the delay of RC. Although performing comparably to gemcitabine-cisplatin in terms of tumor downstaging, the ddMVAC provided significant improvement in overall and cancer-specific survival shaping the long-term outcome of the entire NAC cohort. This remains in contrast to significant part of studies focusing on NAC efficacy, which have either not disclosed the type of cytotoxic regimens used, or analyzed a spectrum of different chemotherapy protocols, hence the observed effect on survival might be a resultant of multiple confounding variables [9, 13, 14, 17]. Our findings are in keeping with observations from Zargar et al. who reported on superior efficacy of ddMVAC over gemcitabine-cisplatin in their retrospective, multicenter analysis conducted in a population of 319 patients with locally advanced T3-T4a MIBC [19]. Whilst the authors found higher response rate and lower risk of all-cause and cancer-specific mortality in subjects treated with ddMVAC in this high-risk cohort, our study corroborated superior efficacy of ddMVAC over a broader range of local stages. Peyton et al. observed a higher proportion of complete remissions and higher probability of downstaging with ddMVAC compared to gemcitabine-cisplatin protocol, however reported difference in survival has not reached statistical significance, likely because there were only 14% of ddMVAC-treated patients in this cohort [10]. Further evidence supporting superiority of MVAC protocol over gemcitabine-cisplatin in terms of survival originate from already cited meta-analysis by Yin et al. who found significantly decreased OS in patients treated with the latter. The study made no distinction between the classical and dose-dense MVAC. Moreover, the survival benefit was overemphasized by ‘contamination’ of gemcitabine cohort with patients who received gemcitabine-carboplatin, hence after exclusion of this subgroup the difference in survival was no longer significant [3]. The carboplatin activity against urothelial cancer is known to be inferior to that of cisplatin, however this agent is occasionally chosen for NAC, owing to its lower toxicity and lower demand for kidney function, regardless of paucity of evidence supporting its use for this indication [20, 21]. Our observation confirms that gemcitabine-carboplatin performs poorly in the neoadjuvant setting, as it failed to generate any significant response rate.

We acknowledge several limitations of this study originating from its retrospective and non-randomized design, lack of standardization of NAC administration, relatively small patient population, and comparison with largely historical surgical cohort which may lead to overemphasis of survival advantage. The relatively short median follow-up only allowed for evaluation of 3-year survival rates. The gemcitabine-cisplatin regimen was administered to fewer patients and was found to provide inferior survival outcome to ddMVAC whereas performing comparably in terms of tumor downstaging. These results might partly be attributed to patients’ selection, as some receiving gemcitabine-platinum combinations could have been considered unfit for more aggressive chemotherapy. Furthermore, COVID-19 related deaths have occurred in our cohort over the last year, affecting exclusively patients from the NAC group, even though they were no longer actively treated at the time of contracting SARS-CoV2 infection. There is sparse evidence on how COVID-19 affects cancer survivors. Carreira et al. evaluated the course of COVID-19 infection in more than 108,000 cancer survivors, including 7712 with bladder cancer. At five years after diagnosis, 62.7% of survivors had at least one comorbidity associated with severe COVID-19 outcomes, while 37.3% had two or more [22]. These findings, along with survival outcomes from our study emphasize, that bladder cancer survivors are at significant risk of concurrent mortality due to burden of their underlying comorbidities. Taking above into consideration, we performed competing risk regression analysis which validated the overall survival improvement with ddMVAC-based NAC. We hypothesize that COVID-19 pandemic-caused alteration of concurrent mortality structure, healthcare disruption and associated disturbance in cancer treatment delivery, challenge the evaluation of long-term efficacy of treatment modalities for cancer [23]. Furthermore, it raises questions on significance of study endpoints and draws attention to more disease-specific outcome measures.

## Conclusions

This single-center retrospective study indicates that multidisciplinary treatment of MIBC involving NAC and RC provides superior outcomes to upfront surgery in terms of local disease control and survival. The ddMVAC protocol was correlated with improved overall and cancer-specific survival compared to gemcitabine-cisplatin. Given the limitations of our study further research, including randomized control trials is needed to validate superiority of ddMVAC over other cytotoxic regimens. Nonetheless, the urologic community should strive to maximize the utilization of NAC amongst cisplatin-eligible patients.

## Supporting information

S1 Table Univariable, multivariable and propensity-weighted regression analysis of factors predicting downstaging (<pT2N0). (XLSX)

S2 Table Univariable, multivariable and propensity-weighted regression analysis of factors predicting complete (pT0N0) pathological response. (XLSX)

S3 Table Univariable, multivariable and propensity-weighted Cox PH model for non-cancer death. (XLSX)

S4 Table Univariable, multivariable and propensity-weighted Cox PH model for cancer death. (XLSX)

S5 Table Univariable, multivariable and propensity-weighted competing risk model for cancer death. (XLSX)

S1 Dataset Study source dataset. (XLSX)
